# The Arch Electrode: A Novel Dry Electrode Concept for Improved Wearing Comfort

**DOI:** 10.3389/fnins.2021.748100

**Published:** 2021-10-18

**Authors:** Beatriz Vasconcelos, Patrique Fiedler, René Machts, Jens Haueisen, Carlos Fonseca

**Affiliations:** ^1^Departamento de Engenharia Metalúrgica e de Materiais, Faculdade de Engenharia, Universidade do Porto, Porto, Portugal; ^2^CEMUC – Department of Mechanical Engineering, University of Coimbra, Coimbra, Portugal; ^3^Institute of Biomedical Engineering and Informatics, Technische Universität Ilmenau, Ilmenau, Germany; ^4^Department of Neurology, Biomagnetic Center, Jena University Hospital, Jena, Germany; ^5^LAETA/INEGI, Institute of Science and Innovation in Mechanical and Industrial Engineering, Porto, Portugal

**Keywords:** biopotential measurement, electrodes, biosignals, electroencephalography, electrode-skin impedance, additive manufacturing, electroless plating

## Abstract

Electroencephalography (EEG) is increasingly used for repetitive and prolonged applications like neurofeedback, brain computer interfacing, and long-term intermittent monitoring. Dry-contact electrodes enable rapid self-application. A common drawback of existing dry electrodes is the limited wearing comfort during prolonged application. We propose a novel dry Arch electrode. Five semi-circular arches are arranged parallelly on a common baseplate. The electrode substrate material is a flexible thermoplastic polyurethane (TPU) produced by additive manufacturing. A chemical coating of Silver/Silver-Chloride (Ag/AgCl) is applied by electroless plating using a novel surface functionalization method. Arch electrodes were manufactured and validated in terms of mechanical durability, electrochemical stability, *in vivo* applicability, and signal characteristics. We compare the results of the dry arch electrodes with dry pin-shaped and conventional gel-based electrodes. 21-channel EEG recordings were acquired on 10 male and 5 female volunteers. The tests included resting state EEG, alpha activity, and a visual evoked potential. Wearing comfort was rated by the subjects directly after application, as well as at 30 min and 60 min of wearing. Our results show that the novel plating technique provides a well-adhering electrically conductive and electrochemically stable coating, withstanding repetitive strain and bending tests. The signal quality of the Arch electrodes is comparable to pin-shaped dry electrodes. The average channel reliability of the Arch electrode setup was 91.9 ± 9.5%. No considerable differences in signal characteristics have been observed for the gel-based, dry pin-shaped, and arch-shaped electrodes after the identification and exclusion of bad channels. The comfort was improved in comparison to pin-shaped electrodes and enabled applications of over 60 min duration. Arch electrodes required individual adaptation of the electrodes to the orientation and hairstyle of the volunteers. This initial preparation time of the 21-channel cap increased from an average of 5 min for pin-like electrodes to 15 min for Arch electrodes and 22 min for gel-based electrodes. However, when re-applying the arch electrode cap on the same volunteer, preparation times of pin-shaped and arch-shaped electrodes were comparable. In summary, our results indicate the applicability of the novel Arch electrode and coating for EEG acquisition. The novel electrode enables increased comfort for prolonged dry-contact measurement.

## Introduction

Electroencephalography (EEG) is a brain monitoring modality and measures the electrical potential distribution on the scalp resulting from brain activity. EEG is widely used for medical diagnostics, e.g., in epilepsy, sleep disorders, coma, anesthesia, and stroke (Niedermeyer and Lopes da Silva, 2005; Puce and Hämäläinen, 2017; Seeck et al., 2017). In the last two decades, its applications extended to an ever-increasing number of areas that include monitoring of motor rehabilitation (Comani et al., 2015; Renton et al., 2017), neurodevelopment in preterm infants (Hayashi-Kurahashi et al., 2012; Berchicci and Comani, 2015; Khazaei et al., 2021), cognitive decline including Alzheimer’s (Paulsen et al., 2012), professional sports training (Cheron et al., 2016; di Fronso et al., 2019), mental states (Al-Barrak et al., 2017; di Flumeri et al., 2019), microstates (Michel and Koenig, 2018), sleep states (Khazaei et al., 2021), cognitive load (Ortiz et al., 2020), and brain computer interfaces (BCI) (Miranda et al., 2015; Stegman et al., 2020). Most new applications require specific characteristics of the EEG equipment so their full potential can be explored in real-life, mobile environments. These requirements include portability, easy and fast setup, wearing comfort, and unobtrusiveness. To date, these specifications have not been fully met, particularly regarding the easy and reliable signal transfer at the scalp-electrode interface under mobile conditions.

Silver/Silver-Chloride (Ag/AgCl) electrodes used with a conductive electrolyte gel for a long time have been the gold standard for every kind of (non-invasive) EEG signal recording due to their reliable electrochemical characteristics, and low level of intrinsic noise. Common limitations like high cleaning effort and limited recording time due to gel drying, have been addressed, e.g., in recent developments of novel hydrogels (Pedrosa et al., 2017). Yet, gel-based EEG suffers from important limitations related to the gel use, e.g., a long preparation time requiring specifically trained technical personnel, risks of allergic reactions, and electrode short-circuits due to gel spreading (Niedermeyer and Lopes da Silva, 2005; Kleffner-Canucci et al., 2012). Finally, gel-based electrode caps cannot be self-applied and the user has to thoroughly wash the head after the exam.

Novel electrode technologies are critical directions of research for signal quality improvement, rapid, and prolonged biopotential measurements. Screen- or flex-printed dry composite electrodes have been proposed and successfully validated for applications with low hair density, including electrocardiography (Chlaihawi et al., 2018), electromyography (Pani et al., 2019) and below-hairline EEG (Bleichner and Debener, 2017; Blum et al., 2020). Furthermore, design alternatives have been proposed to overcome the problems of the electrolyte gels at hairy areas of the head. Micro-spiked electrodes were proposed (Griss et al., 2002; Fu et al., 2020), where a micro-needles array perforates the stratum corneum insulating skin layer, to achieve a reliable contact and a low-impedance path without gel. The reported performance is very close to that of commercial gel-based Ag/AgCl electrodes. However, 5% of the spikes break during the exam, eventually remaining embedded in the epidermis. Hence, this must be considered an invasive technique not free from infection and inflammatory risks. In the quasi-dry (or semi-dry) electrode concept an electrolyte-based, low impedance contact is obtained without dirtying the scalp by dispensing a small amount of a hydrating fluid specifically at the electrode-scalp contact point in a controlled way. Wick materials are used for the electrodes, whose working principle is close to that of a felt pen (Li et al., 2016; Peng et al., 2016; Pedrosa et al., 2018). Alternatively, a cavernous structure holds a small amount of electrolyte, dispensed by, e.g., deformation under pressure during application (Mota et al., 2013). For a recent comprehensive review of semi-dry electrodes, please refer to Li et al. (2020). The signal quality of these electrodes proved to be similar to conventional Ag/AgCl gel-based electrodes, even reaching a recording autonomy higher than those (6–8 h), due to the liquid reservoir on the back of the electrode. However, comfort is still a considerable issue, due to the required adduction and stiffness of the materials. Translation of this technology into a functional, multi-electrode cap is costly and self-application by a user may be error-prone.

Dry electrodes represent a radically different approach, characterized by the absence of electrolyte fluids at the electrode-scalp interface (Grozea et al., 2011; Salvo et al., 2012; Fiedler et al., 2015; Mullen et al., 2015; di Flumeri et al., 2019). For this reason, the coupling relies exclusively on the skin-to-electrode interface and mechanical force exerted thereat. Several design approaches have been developed for the electrode to pass through the hair layer and ensure reliable adduction to the scalp, the main problem faced by dry electrodes. Salvo et al. (2012) and Fiedler et al. (2015) proposed a pin-like approach. Mullen et al. (2015) introduced the spider-shaped sensor, a multi-tip electrode with the ability to deform upon application of the adduction pressure. Grozea et al. (2011) developed a brush-like electrode. Some of these designs are already available as commercial products in the form of multi-electrode caps and headsets (Fiedler et al., 2015; Mullen et al., 2015). The main advantages of dry electrode based systems are to (i) significantly decrease the preparation time for the exam (5–10 min vs. 30–40 min), (ii) avoiding the need for a technician, enabling self-application by the user, and (iii) eliminating extensive hair/scalp preparation or gel application, thus also (iv) minimizing post-exam cleaning requirements. Therefore, dry electrodes represent an important step in the pursuit of a plug-and-play, portable and unobtrusive EEG system.

Yet, dry electrodes are not free of limitations, namely a much higher electrode-skin interfacial impedance (10–20 kΩ vs. 200–1,000 kΩ) (Grozea et al., 2011; Salvo et al., 2012; Fiedler et al., 2015; Mullen et al., 2015), which translates in the signal acquisition to be more prone to be contaminated by environmental noise and more susceptible to movement artifacts (Oliveira et al., 2016; di Fronso et al., 2019; Kam et al., 2019; Marini et al., 2019). Another important disadvantage is the limited comfort, which is often reported to be lower than for gel-based electrodes, thus limiting the total wearing time. The discomfort is worse for solid metal pin electrodes (Xu et al., 2017) and it can be improved by the use of polymeric (semi-)flexible (Fiedler et al., 2015; Mullen et al., 2015), or spring-loaded pins (Hinrichs et al., 2020; Kimura et al., 2020). However, existing pin designs require a compromise between (a) flexibility of the (soft) pins to avoid pressure spots and increase wearing comfort, and (b) stiff pins to ensure a reliable, easy, and rapid mechanical coupling necessary for optimal EEG signal quality (Fiedler et al., 2018).

An alternative to the pin-based design was recently proposed (Lee et al., 2015; Kim et al., 2019), with the silver-based Arch/Comb electrode. In this approach the scalp contact is achieved by a set of parallel, equally spaced arches instead of pins, with the hypothesis that the arches may provide a larger contact surface, consequently limiting local pressure spots and improving the wearing comfort. The authors concluded that this electrode not only displays lower contact impedances but indeed provides more comfort than pin-based electrodes and it enables the acquisition of more accurate EEG signals. On the disadvantages, this electrode was fabricated from sterling silver. It is certainly expensive, heavy, and most likely not a viable commercial solution, despite the interesting concept. Moreover, solid metals are not capable of adapting to different head shapes and sizes, consequently limiting long-term wearing comfort.

We present a novel approach of an Arch-shaped electrode, fabricated using additive manufacturing of a thermoplastic polyurethane (TPU) substrate, chemically coated with an Ag/AgCl film. This approach accounts for a lighter, cheaper, and more comfortable electrode, given that the arches have the ability to adapt to the scalp. We present a full electrochemical, mechanical and multi-channel applicability study of the novel Arch dry electrode in comparison to both conventional gel-based electrodes and Multipin-shaped dry electrodes.

## Materials and Methods

### Electrodes

**FIGURE 1:**
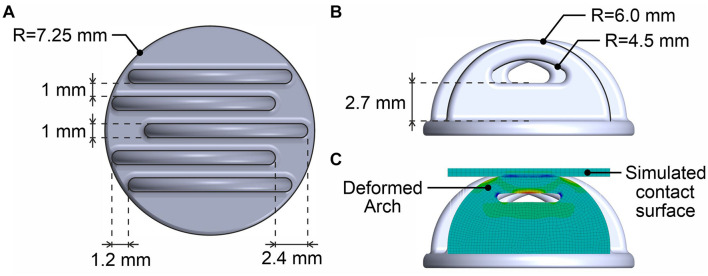
3D model of the Arch electrode design: **(A)** Primary dimensions in top view, **(B)** Side view, and **(C)** Illustration of the deformation under application of a radial force to the tip of the arch.

The Arch electrode comprises five equally spaced and equally sized arches with a width of 1 mm, an outer radius of 6 mm, an inner radius of 4.5 mm, and a 1 mm spacing in-between. The arches are arranged on one common baseplate, with an alternating shift of 1.2 mm and 2.4 mm. The shift reduces the tilting of the electrodes on the head of the user. The lower half of the arch is filled to increase the stability of each arch against sideway bending. On the contrary, the upper 40% of the arch is hollow to allow radial deformation under the application of force. A scheme of the complete Arch electrode design and dimensions are provided in Figures 1A,B. Figure 1C shows the setup for the deformation study for a single arch, deformed by the application of a radial force at the top. The simulation is similar to the conditions when adducting the electrode to the head surface within an EEG cap and illustrates the basic principle of the Arch electrode. The deformation of the arch under radial forces is intended to increase the contact surface and thus reduce pressure, consequently improving the wearing comfort for the user by avoiding excessive local pressure spots.

The electrode substrate was manufactured using a TPU filament with shore hardness A 93 (TPU 93, German RepRap GmbH, Feldkirchen, Germany), and a commercial fused deposition modeling (FDM) printer (N2 Plus, Raise 3d, Irvine, United States) with a lateral resolution of 200 μm and a layer thickness of 75 μm. Subsequently, the electrically non-conductive substrate was coated using a multi-phase electroless plating with Silver/Silver-Chloride (Ag/AgCl) by an adapted process based on Vasconcelos et al. (2018): (1) Cleaning of the substrate by exposure to Isopropanol and subsequently distilled water in an ultrasonic bath for 5 min each. (2) Increasing the polymer’s surface affinity to Ag by immersion in a 15% PVP:DMSO solution for 30 s. (3) Functionalization of the surface by adsorption of Ag^+^ followed by its reduction to Ag^0^. (4) Silver plating using an adapted Tollens reagent. (5) Cleaning, drying, and chlorination.

During the *in vivo* application study, the novel Arch electrodes are compared to (a) commercial, sintered Ag/AgCl ring electrodes for gel-based application (B10-HS, Easycap GmbH, Herrsching, Germany), and (b) AgCl coated Multipin dry electrodes with Shore hardness A98, according to Fiedler et al. (2015). A summary of all compared electrode’s design, material composition, and production process is provided in Table 1.

**TABLE 1:** Overview of the three compared electrode types with material and manufacturing details.

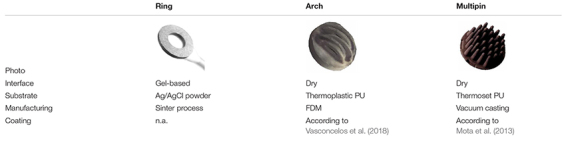

### Electroencephalography Caps

Based on the international ten-twenty system for electrode placement (Klem et al., 1999), 21 electrodes of each of the three electrode types were integrated into separate EEG caps. All electrodes were equipped with custom micro-coaxial cables and connectors to be connected to the EEG amplifier.

The gel-based Ag/AgCl ring electrodes have been integrated into a commercial semi-rigid EEG cap (EC-40, Easycap GmbH, Herrsching, Germany). Both the novel dry Arch electrodes and the Multipin dry electrodes have been integrated into custom-made flexible fabric caps. The custom cap’s single-layer fabric is composed of a combination of cotton, elastane, and polyamide and is produced by flat knitting (Wunder et al., 2018). For each of the caps, only a medium-size sample was assembled for head circumferences ranging from approx. 56–59 cm. For the two compared dry electrode types, identical cap fabric material and cut were used to avoid related differences in electrode adduction across the measurement conditions.

**FIGURE 2:**
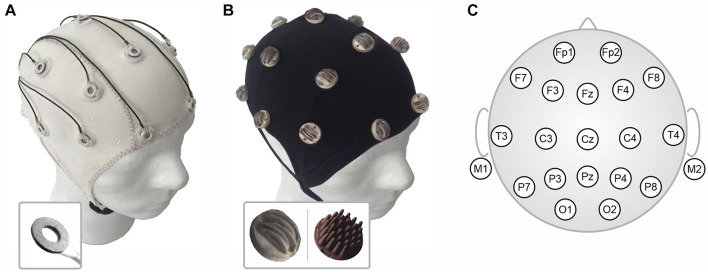
Assembled electrode caps: **(A)** Commercial fabric cap with sintered Ag/AgCl ring electrodes for gel-based application (cap shown from the outside), **(B)** Custom-made flexible fabric cap for integration of either Arch (inset left) or Multipin (inset right) dry electrodes (cap shown turned inside-out), and **(C)** Scheme of the 21-channel clinical layout according to the international ten-twenty system used for the *in vivo* EEG recordings.

A scheme of the electrode setup along with photographs of the assembled caps and electrode types are shown in Figure 2.

### Electrochemical Characterization

A G300 potentiostat (Gamry Instruments Inc., Warminster, United States) was used for all electrochemical electrode characterizations, including measurement of the open circuit potential (OCP), electrochemical impedance spectroscopy (EIS), and electrochemical potential noise (EPN). The hardware was controlled by the Gamry framework software (OCP and EIS) and the Gamry ESA410 electrochemical signal analyzer (EPN). All electrochemical measurements were performed with the electrodes immersed in a 0.9% sodium chloride solution (Ref. 31434, Sigma Aldrich, St. Louis, United States). Four and five Arch electrode samples were tested for OCP and interfacial impedance, respectively. The electrochemical cell was maintained inside a Faraday cage in all the tests in order to reduce external noise.

The OCP was recorded for each electrode during 3,600 s at 0.5 samples/s, against an Ag/AgCl reference electrode (XR 300, Radiometer Analytical, Hach, United States). EIS data was acquired in the potentiostatic mode from 66 mHz to 66 kHz (1 point per decade), by applying a sinusoidal voltage with 5 mV amplitude (rms). The Ag/AgCl reference electrode and a platinum wire were used as reference and counter electrodes, respectively. The standard error associated with the impedance values was calculated by using the standard deviation and the Student’s T-distribution with a 95% confidence level. EPN was acquired between two separate sample pairs of Arch electrodes for 15 min with a sampling frequency of 1 kHz. The calculation of the Power Spectral Density (PSD) was performed by using the ESA410 software with a Hanning smoothing window. The rms value of noise was calculated after detrending the data with a 4th-degree polynomial function.

### Mechanical Wearing Tests

For testing of the durability of the TPU coating to strain and bending, dedicated samples were produced and exposed to respective tests.

A silver-plated TPU sample (7 × 2.5 cm) was strained at 10 mm/min using a tensile testing machine (EZ Test, Shimadzu Corp., Kyoto, Japan). Its electrical resistance was simultaneously measured with a voltmeter (DVM890, Velleman NV, Gavere, Belgium) connected to both ends of the sample.

Similarly, the influence of material fatigue imposed by bending was evaluated by measuring the electric resistance with a voltmeter (DVM890) at opposite diameter points of samples conformed to steel cylinders of 2.5, 5, and 7.5 mm radius before (R0) and after (R) every 50 cycles of bending.

Adhesion of the silver coating to the TPU substrate was measured with a tensile testing machine (EZ Test, Shimadzu Corp., Kyoto, Japan) at a crosshead speed of 50 mm/min while performing a T-Peel adhesive peel testing by peeling off a double-sided adhesive (tesa POWERBOND^®^, 19 mm width, 2 mm thick), according to ASTM D1876.

### *In vivo* Measurements

Ten male and five female healthy volunteers with an average age of 30 ± 4 years, an average head circumference of 57 ± 1 cm, and a hair length ranging from 0.5 cm to 42 cm participated in the *in vivo* study. All volunteers reported a healthy skin state and no prior record of drug abuse, neurological or psychological disorders. The study complied with the ethical standards outlined in the Declaration of Helsinki and was approved by the local Ethics Committee. All volunteers provided written informed consent before they participated in the study.

Electroencephalography and electrode-skin impedance recordings were performed with a referential biosignal amplifier (EE-225 eego^TM^ amplifier, ANT Neuro B.V., Hengelo, Netherlands) using active shielding applied to the coaxial electrode cables.

The three caps and electrode types were tested sequentially on the same day. A break of 1 h was ensured between the different electrode cap tests, to allow the volunteer’s skin to recover and further minimize cross-influences. For each of the measurements, reference and patient ground electrodes were placed at the right and left mastoids, respectively. Prior to the application of the reference and patient ground electrodes, the skin at the electrode positions was cleaned using medical alcohol pads (B. Braun Melsungen AG, Melsungen, Germany). Gel-based, sintered Ag/AgCl ring electrodes (B10-HS, Easycap GmbH, Herrsching, Germany) in combination with commercial electrolyte gel (Electro-Gel, Electro-Cap International Inc., Eaton, United States) were used in case of the reference and ground electrodes. Furthermore, medical adhesive tape (Leukopor Ref. 0247100, BSN medical GmbH, Hamburg, Germany) was used to fixate these electrodes at the mastoids.

All volunteers were asked to comb their hair and distribute it evenly on the head prior to each cap application. No skin cleaning or abrasion was performed at the 21 measurement electrode positions. Only for the measurements with gel-based electrodes, electrolyte gel (Electro-Gel, Electro-Cap International Inc., Eaton, United States) was applied between electrodes and skin, ensuring an electrode-skin impedance level of 30 kΩ or lower. In line with our previous publications (Fiedler et al., 2015; di Fronso et al., 2019), no impedance threshold was defined for the dry electrodes but the operators were asked to optimize electrode-skin contact individually until no further improvement was perceived possible. In the case of the Arch electrodes, preparation included the adaptation of the arch orientation in line with the individual hair orientation of the volunteers by lifting and rotating the electrode.

Our previously established validation paradigm was used for the assessment of differences in signal characteristics across the electrode types. Consequently, for each volunteer and each electrode type, three EEG tests have been recorded: resting state EEG with open eyes, resting state EEG with closed eyes (alpha activity), and a pattern reversal visual evoked potential (VEP) comprising 150 trials. Perceived wearing comfort evaluation was reported using the Scott and Huskisson pain scale, ranging from 1 to 10 (Scott and Huskisson, 1976). The wearing comfort was assessed after initial cap application as well as after approx. 30 min and 60 min of wearing the cap.

For both dry electrode caps, an additional re-application test was performed after the last EEG recording. Therefore, the dry electrode caps were taken off completely. After combing the hair and a subsequent break of 10 min, the caps were applied again and the respective re-application time was determined in line with the original application procedure.

### Signal Processing and Analysis

The *in vivo* data acquisition was performed using eego^TM^ software (ANT Neuro BV, Enschede, Netherlands) with a sampling rate of 1024 samples/sec. Postprocessing and analysis of the data were performed using custom MATLAB scripts (The Mathworks, Natick, United States).

The investigated frequency band was limited to standard EEG between 1 Hz and 40 Hz, applying a Butterworth bandpass with respective cut-off frequencies. Subsequently, the data were manually reviewed, identifying bad channels and excluding them from further analysis. After filtering and exclusion of bad channels, the remaining channels have been re-referenced to common average reference. Analysis epochs of 30 s length were extracted. The Welch estimation of the PSD was calculated for resting state EEG both with closed and open eyes.

For the analysis of the VEP results, 150 stimulation trials were averaged per volunteer. Subsequently, the global field power (GFP) was calculated (Fiedler et al., 2015). The N75 and P100 peak latencies and power were determined in the GFP and the signal-to-noise ratio for each peak was calculated. The signal was defined as the peak power value in the GFP, while the noise was defined as the power at the time of the stimulus onset.

The statistical significance of differences between the electrode types in terms of electrode-skin impedance and comfort was tested using Wilcoxon-Mann-Whitney *U* tests at a Bonferroni-corrected alpha level of 0.017.

## Results

### Electrochemical Characterization

**FIGURE 3:**
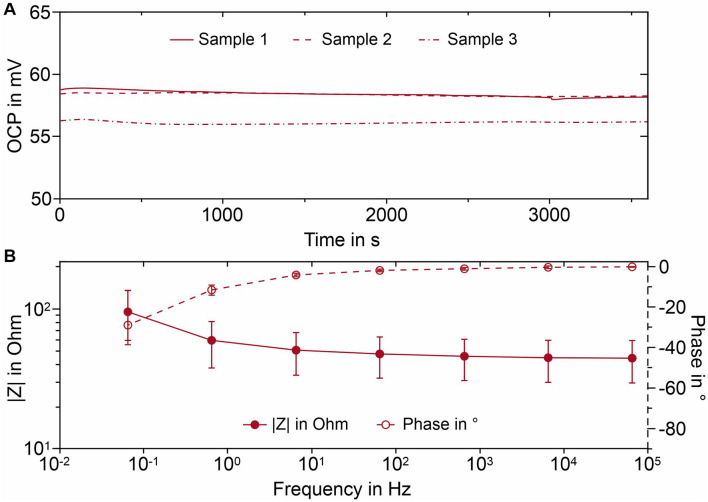
Electrochemical characterization of the AgCl coated Arch electrode samples immersed in 0.9% sodium chloride solution: **(A)** OCP curves for three exemplary electrodes; **(B)** Average Bode plot and standard error. Whiskers indicate the 95% confidence interval.


The OCP at the interface between an electrode and the electrolyte has an important impact on the quality of the recorded signal. A high OCP drift and offset may hamper the recording of slow potential waves and, in the worst case, lead to saturation of the amplifier input. The plot of Figure 3A represents the OCP curves for three exemplary Arch electrodes. It is apparent that the Arch electrodes display a low potential drift and good potential reproducibility. This is due to the electrochemical reaction according to eq. 1 (Huigen et al., 2002):

(1) $$A\,g+C\,l^{\text{-}}\,⇄A\,g\,C\,l+e\,l\,e\,c\,t\,r\,o\,n$$

The reaction takes place at the electrode/electrolyte interface that rapidly reaches the equilibrium state, thus locking the interfacial potential.

Electrochemical impedance spectroscopy (EIS) is a powerful technique to study the electrochemical properties of a metal-solution interface (Orazem and Tribollet, 2008) because it enables the determination of the impedance of the interface in a broad frequency range. A stable and low interfacial impedance in the frequency range of interest is essential for accurate biosignal monitoring (Searle and Kirkup, 2000). The Bode plot of the interface between Arch electrode and sodium chloride solution is reported in Figure 3B. In the high frequency region (66 Hz–66 kHz) the impedance remains essentially constant (≈ 45 Ω) and the signal phase is about −2°. Consequently, the interface is predominantly characterized by resistive characteristics. This contribution is related to the resistance to charge transfer across the interface, related to the equilibrium (cp. Eq. 1). For frequencies below 66 Hz there is a phase shift and the impedance starts to increase, being an indication that the measured impedance receives a capacitive contribution, coming from interfacial charge accumulation at the interface (Pedrosa et al., 2015). However, the impedance remains below 100 Ω throughout the investigated frequency band, even for the lowest investigated frequency of 66 mHz, thanks to the very large interfacial area displayed by the AgCl layer.

**FIGURE 4:**
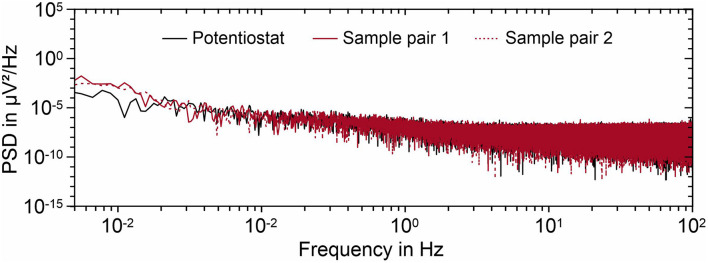
PSD of the recorded noise for two Arch electrode sample sets immersed in 0.9% sodium chloride solution. The potentiostat noise is also displayed for comparison. The rms values of noise are reported in the graph.

The EPN analysis between two independent electrode sample pairs was performed in order to measure the noise contribution of the interface between the Ag/AgCl and the sodium chloride solution to the total measured noise. The PSD of noise measured for two Arch electrode sample pairs are reported in Figure 4, together with the electrical noise measured with the potentiostat inputs short-circuited. It can be concluded that the rms value of electrochemical noise is below 0.01 μV^2^/Hz and it only differentiates from hardware noise for frequencies below 20 mHz.

### Mechanical Wearing

**FIGURE 5:**
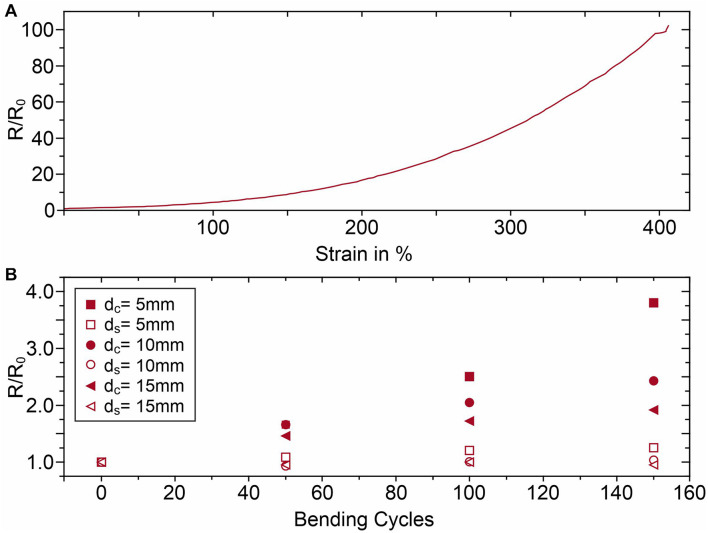
Electrical resistance changes of the Ag coating of the TPU samples due to **(A)** strain tests, and **(B)** bending cycle tests; d_*c*_ is the cylinder diameter used for compression and d_*s*_ is the cylinder diameter used for stretching.

**FIGURE 6:**
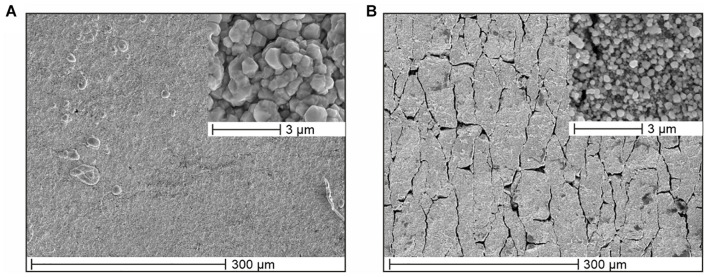
SEM micrographs of silver coated TPU samples **(A)** before, and **(B)** after 400% strain tests with insets at increased magnification.

A TPU substrate was chosen for the manufacturing of the Arch electrodes due to its elastomeric nature and inherent flexibility, to reduce the patient discomfort while wearing the cap and enhance the electrode/skin contact area. However, silver coatings don’t share the same flexibility as the polymeric substrate, which can lead to discontinuities on the films and consequently to electrical conductivity reduction or failure. Nevertheless, the electroless plated silver coating on the Arch electrodes can withstand a maximum of 400% deformation before electrical failure, as seen in Figure 5A. Figure 6 shows the SEM micrographs of silver coated TPU samples before and after the 400% strain test. It is apparent that, due to lower stretchability of the silver film compared to TPU, a network of fractures forms on the film, perpendicular to the direction of the applied stress. Moreover, the silver grain size decreases after the strain test (see insets for both conditions). This may indicate that the grains have been broken as well. However, even for such high values of deformation, the sample remains conductive, what may be ascribed to the silver that deposits several microns beneath the film, into the polymer, firmly anchoring the film to the substrate (Vasconcelos et al., 2018).

Furthermore, the fatigue tests evidenced that the silver coating is more sensitive to compressive forces, see Figure 5B, as the normalized resistance has a greater tendency to increase as the bending diameter decreases. In the worst case, i.e., 150 bending cycles for the 5 mm diameter cylinder, the resistance increases from 0.9 to 3.2 Ω. However, after both strain deformation at 400% and fatigue tests, the samples return to their original shape and initial resistance R_0_, after approximately 5 min. This means that the silver coating can adapt to the TPU substrate’s elasticity due to its adhesion to the substrate being >8.5 N/cm.

### Preparation and Comfort

The preparation times for each electrode cap, measured from the time of initial cap application until the start of the first EEG recording, were 22 ± 4 min (gel-based), 15 ± 3 min (Arch), and 5 ± 1 min (Multipin) during the first application. For the second application of the Arch electrode caps, the preparation time decreased to 6 ± 2 min, whereas the preparation time for the Multipin electrode cap remained constant at 5 ± 1 min.

**FIGURE 7:**
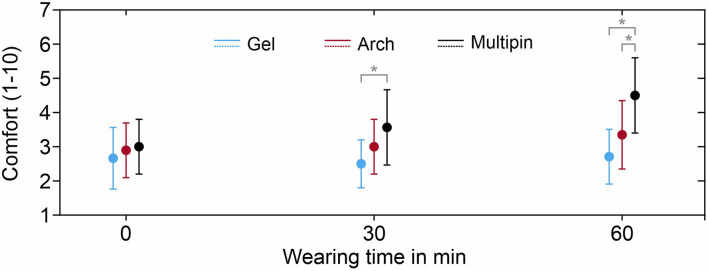
Average comfort evaluation of the three electrode types after application (0 min), 30 min, and 60 min, on a scale ranging from 1 (max. comfort) to 10 (pain, requirement to take the cap off). Whiskers indicate standard deviation. Asterisks indicate statistically significant differences.

The evolution of the comfort evaluations during the complete measurement time is shown in Figure 7. The best comfort evaluation was achieved using the gel-based cap with evaluation values of 2.6 ± 0.9 (0 min), 2.5 ± 0.7 (30 min), and 2.7 ± 0.8 (60 min). The comfort evaluation for the Arch electrodes was 2.8 ± 0.8 after application, compared to 3.1 ± 0.9 for the Multipin cap. The strongest decrease of comfort was determined for the Multipin electrodes as well, with the lowest comfort of 4.5 ± 1.1 after 60 min. While no significant difference in comfort between the electrode types was evident directly following the application, already after 30 min, the gel-based electrodes showed a significantly better comfort than the Multipin electrodes (*p* = 0.0026), whereas the difference between gel-based and Arch electrodes was not significant (*p* = 0.1396). After 60 min of wearing, the comfort of gel-based vs. Multipin (*p* = 0.0002) and Arch vs. Multipin (*p* = 0.0114) differed significantly, whereas the difference gel-based vs. Arch resulted in *p* = 0.0498 and thus above the Bonferroni-corrected significance threshold of *p* = 0.017. The average evaluations remained below 5 for all electrode types, and therefore within the non-painful range of the scale ranging from 1 to 10.

### Electrode-Skin Impedance and Channel Reliability

**FIGURE 8:**
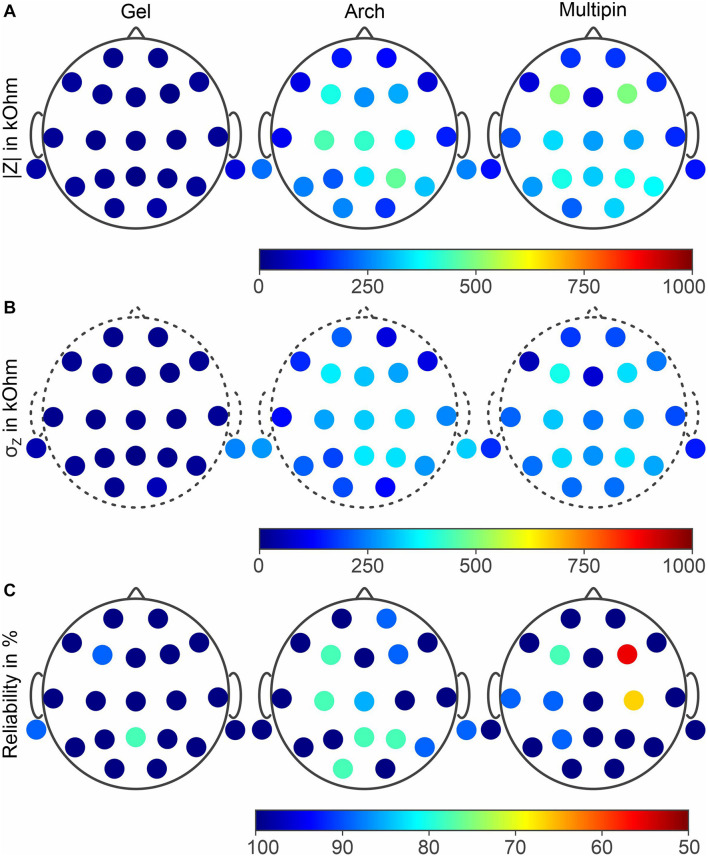
Grand averages of **(A)** the electrode-skin impedances for all three compared electrode types, **(B)** standard deviation of the impedance measurements, and **(C)** channel reliability in 45 EEG recordings. Results for gel-based (left), Arch (center) and Multipin (right) electrodes.

The grand average electrode-skin impedances of the three compared electrode types are shown in Figure 8A (mean) and Figure 8B (standard deviation). The means across all electrode positions are 22 ± 18 kΩ (gel-based), 257 ± 118 kΩ (Arch electrode), and 264 ± 125 kΩ (Multipin). No statistically significant difference between the Arch electrode and Multipin electrode impedances could be determined (*p* ≥ 0.0225), whereas gel-based vs. Arch (*p* ≤ 0.0161) and gel-based vs. Multipin (*p* ≤ 0.0039) differed significantly. For both dry electrode types, lower impedances are visible in the frontal and temporal areas, while impedances in the central and parietal head regions are increased. An increased standard deviation and thus impedance level variability is evident for the mastoid electrode positions during the gel-based measurements.

The relative channel reliability indicates the ratio between recordings in which a given channel was not marked as a bad channel and the overall number of recordings (15 volunteers × 3 tests = 45 recordings). The results for the three electrode types are shown in Figure 8C. For the gel-based recordings, channel reliability below 100% resulted for channels F3, M1, and Pz. For the Arch dry electrodes, channel reliability below 100% is evident at positions Fp2, F3, F4, C3, Cz, Pz, P4, P6, M2, and O2. When using Multipin dry electrodes, channels F3, F4, T3, C3, C4, and P3 showed reliability values below 100%. The lowest and the second lowest channel reliability values are also evident for the Multipin dry electrodes at F4 and C4 positions, respectively. The means and standard deviations of the overall reliability were 97.9 ± 5.7% (gel-based), 91.9 ± 9.5% (Arch), and 93.7 ± 12.5% (Multipin).

### Electroencephalography Signal Characteristics

**FIGURE 9:**
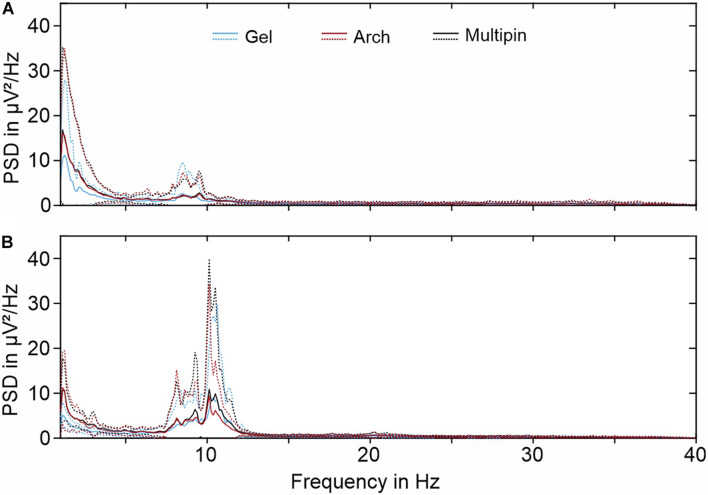
Grand averages of the PSD (Welch estimation) for 30 s of EEG recorded with all three electrode types during **(A)** resting state with eyes open and **(B)** resting state with closed eyes. Solid lines indicate mean values, while dotted lines indicate standard deviation.

The PSD of sequential EEG recordings performed under similar conditions can be used for the comparison of signal characteristics in the frequency domain. The Welch estimation of the PSDs for resting state EEG recordings with open eyes and closed eyes are displayed in Figures 9A,B, respectively. Comparing the two recording conditions (open vs. closed eyes), alpha suppression is clearly evidenced by the reduced power in the alpha band (8–13 Hz). Peak power and peak frequencies in the alpha band are 9.0 μV at 10.4 Hz (gel-based), 9.4 μV at 10.1 Hz (Arch), and 10.9 μV at 10.1 Hz (Multipin).

Overall, the PSD curves of different electrode types are similar. Both the grand average mean values and standard deviations strongly overlay for the three electrode types. However, slightly reduced power of the gel-based recordings can be observed for frequencies below 3 Hz. Similarly, an increased power below 3 Hz is visible for all electrode types in the resting state condition with open eyes.

In addition to the PSD, we determined the offset between the measurement channels and the reference electrode at the beginning of each EEG recording. These offset values can be derived directly from the EEG recordings of the used DC-EEG amplifier. The mean and standard deviation for the electrode types were −0.4 ± 21.8 mV (gel-based), 5.6 ± 39.3 mV (Arch), and 6.3 ± 29.4 mV (Multipin).

**FIGURE 10:**
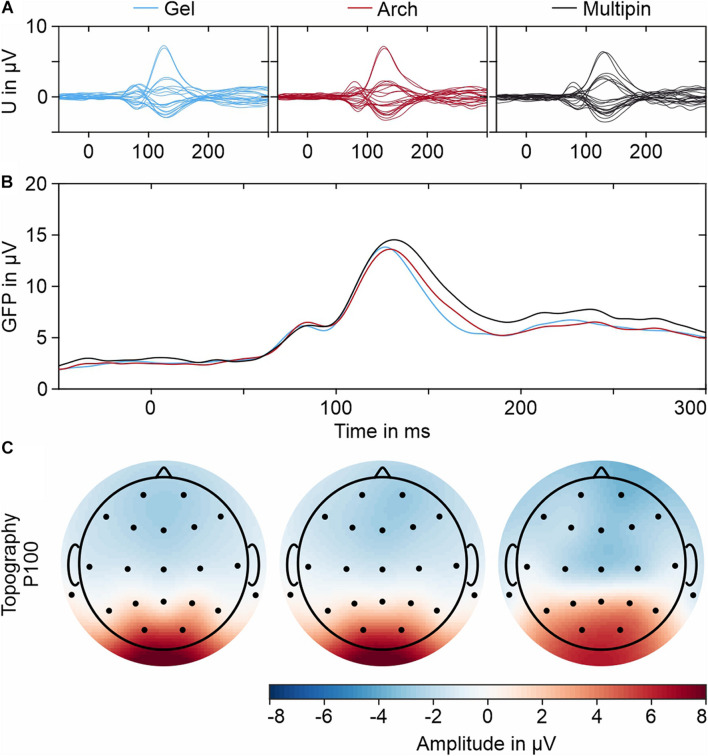
Grand averages of the VEPs recorded with all three electrode types: **(A)** Individual butterfly plots of all 21 EEG channels, **(B)** Overlay plot of the GFPs for all electrodes, and **(C)** Exemplary topographic plots for the P100 peak (determined in the respective GFPs).

In line with the results of the PSD evaluation, the time-domain evaluation of the VEPs shows no considerable differences between the three electrode types. The results in terms of peak latencies, amplitudes, and SNR are listed in Table 2 and displayed in Figure 10. The N75 and P100 of the pattern reversal VEP are clearly pronounced both in the individual butterfly plots and the overlay plot of the GFP shown in Figures 10A,B, respectively.

**TABLE 2:** Quantitative comparison of the N75 and P100 peak latencies, amplitudes and SNR when using gel-based ring electrodes, dry Arch and dry Multipin electrodes.

	**Gel-based Ring**		**Dry Arch**		**Dry Multipin**	
	**N75**	**P100**	**N75**	**P100**	**N75**	**P100**
Latency (ms)	81.3 ± 4.0	128.1 ± 9.1	82.9 ± 3.8	129.1 ± 9.9	81.7 ± 5.0	131.9 ± 8.1
Amplitude (μV)	6.5 ± 2.2	15.0 ± 4.1	6.7 ± 3.1	14.4 ± 3.8	6.6 ± 5.0	16.0 ± 8.1
SNR	3.0 ± 1.0	7.5 ± 3.6	3.0 ± 1.5	6.5 ± 2.6	2.6 ± 1.2	6.3 ± 2.5

*Values represent mean ± standard deviation calculated over the GFP of all 15 volunteers.*

The differences between N75 and the P100 amplitudes and latencies between the electrode types are within intra-individual variability (Fiedler et al., 2014). The SNR is similar for the three electrode types. As visible in Figure 10C, the main features of the topographies of the P100 component of all three caps are similar and show the expected potential distribution.

## Discussion

We successfully developed a polymer-based Arch electrode design and manufacturing technology, combining a specifically optimized Arch electrode shape, additive manufacturing and a new electroless plating technique for Ag/AgCl coated electrodes. We evaluated the electrode’s applicability to EEG acquisition in terms of electrochemical characteristics, mechanical stability of the coating, application time and comfort as well as multichannel EEG signal characteristics in frequency, time, and spatial domain.

The electrochemical characterization highlights the stability of the chemically fabricated AgCl coating when immersed in NaCl solution. The OCP values are highly reproducible and show low drift, in line with other AgCl-based electrodes (Fiedler et al., 2014). The offsets between the individual samples as well as between the samples and the AgCl reference electrode are within a range that allows for recording with state-of-the-art biosignal amplifiers. Similarly, the electrode-electrolyte interfacial impedance is lower than 100 kΩ in the investigated frequency range. The electrochemical noise of the electrode-electrolyte interface is negligible in comparison to the noise spectra of the equipment used. All results of the novel electroless plating for thermoplastic PU are therefore comparable to our findings on the previous plating technique for thermoset PU (Fiedler et al., 2015). In summary, the electrochemical characterization results fulfill the requirements for biosignal acquisition and enable compatibility of the novel Arch electrodes with state-of-the-art amplifiers.

The mechanical stability tests of the electrodes underline that the silver coating adheres well to the TPU substrate and can withstand a strain deformation up to 400% at a strain rate of 10 mm/min before electrical failure. The fatigue tests show a resistance increase from 0.9 to 3.2 Ω which is negligible compared to the interfacial impedance between dry electrodes and skin, typically in the order of several hundred kΩ. When the mechanical stress is released, the samples recover their initial conductivity after a few minutes. Thus, it can be assumed that the Arch electrodes will remain reliable after repeated usage. In case of extreme mechanical stress, care must be taken to perform EEG measurements with some minutes of interval, so that the silver coating on the electrodes has time to conform to the elastic TPU substrate and return to its percolation network.

The results of the *in vivo* EEG recordings prove that the signal quality of the Arch-shaped dry electrodes is comparable both to our Multipin dry electrodes and to gel-based reference electrodes in terms of signal amplitude, power spectral density, and spatial potential distribution. The average channel reliability of the dry setup was 91.9 ± 9.5% which is slightly lower than gel-based electrodes but in the same order of magnitude as the tested Multipin dry electrodes. Electrode failures usually occur in areas with low electrode adduction. Compared to our previous multichannel EEG studies (Fiedler et al., 2015; di Fronso et al., 2019), the reported channel reliabilities are higher, due to the lower electrode number in the present setup as well as due to the per-electrode adaptation during the application and preparation procedure. Low electrode reliability at the mastoid electrodes in all recordings likely results from very low electrode adduction and positioning stability due to the fabric cut. An adapted cut must be used for similar studies in the future to improve adduction and stability of electrodes placed around the ear, especially when eventually extending the electrode layout to the new 25-channel layout for clinical EEG (Seeck et al., 2017) or beyond.

Similar to the electrochemical characterization results, the electrode-skin impedances and EEG channel offset potentials of the electrodes show compatibility with state-of-the-art biosignal amplifiers.

The signal quality in the frequency domain (PSD), time and spatial domain (VEP) showed no considerable differences between the dry and the gel-based electrodes, in line with previous studies of dry AgCl electrodes. The increased spectral power of dry electrodes below 3 Hz was observed similarly in previous studies (Fiedler et al., 2015) and may be related to instabilities at the electrode-skin interface at low frequencies. In combination with the electrochemical characterization, these findings support the conclusion of the developed plating technique to provide adherent and electrochemically stable AgCl coating, fulfilling signal quality requirements for bioelectric sensing applications. In contrast to our previously used coating of thermoset PU, the novel plating technique is specifically developed for thermoplastic PU, and therefore specifically suitable for electrode substrates produced by additive manufacturing.

The Arch electrodes proved to be more comfortable than Multipin electrodes, especially when used for applications of more than 60 min duration. On the other hand, the novel Arch electrodes require individual per-electrode adaptation in terms of aligning electrode rotation and hairstyle of the volunteers. This increased the initial preparation time of the multichannel Arch electrode cap to 15 min, compared to only 5 min for Multipin dry electrodes. However, this preparation time is still lower than that of the gel-based electrode caps with 22 min of preparation on average. Furthermore, when re-applying the Arch electrode caps on the same volunteer, the preparation time of Multipin-shaped and Arch-shaped electrodes was comparable. The initial preparation time may be reduced by ensuring an initial orientation of the electrodes at each head region according to the most common hair orientation (Kawana et al., 2020). Therefore, the Arch electrodes are specifically suited for headsets and head caps intended for repetitive application by the same user, e.g., for BCI and neurofeedback.

Further optimization of the plating technique may focus on cost reduction as well as investigating and improving homogeneity and reproducibility of the coating thickness for serial production. Arch electrode variants with designs (most important height) and flexibility adapted for distinct head regions may be developed, similar to the previously proposed wave-shaped electrodes for forehead and non-hairy head regions (di Fronso et al., 2019). Region-specific electrode designs may further improve the comfort and ease of application. The development of headsets and head caps, specifically designed for applications like BCI and neurofeedback may contribute to decrease the preparation time and improve the adduction of electrodes at central and parietal head regions to increase the respective channel reliability. Dedicated electrode designs may be used for further electrophysiological signals like ECG and EMG (Zhang et al., 2020).

## Conclusion

Our results prove the applicability of the novel silver-coated, polyurethane Arch electrode for EEG acquisition. The novel Arch electrode provides increased comfort especially for repetitive dry-contact biopotential measurement applications and application times longer than 60 min duration. Moreover, beyond the application at hand, the novel electroless plating technique for thermoplastic PU enables easy adaptation of electrode shape to different requirements, including fully individualized electrode shapes for body sensor network applications including multichannel EEG, array electromyography, and electrocardiography.

## Data Availability Statement

The raw data supporting the conclusions of this article will be made available by the authors, without undue reservation.

## Ethics Statement

The studies involving human participants were reviewed and approved by Ethics Committee of the Jena University Hospital, Germany. The participants provided their written informed consent to participate in this study.

## Author Contributions

BV developed the coating technique and performed the coatings as well as the mechanical characterization of the samples. BV and PF prepared, supervised and partially performed the *in vivo* data acquisition. PF performed the *in vivo* data processing and analysis. RM manufactured the electrode substrates. BV, PF, and CF wrote the first draft of the manuscript. All authors contributed to the conception and design of the study, manuscript revision, read, and approved the submitted version.

## Conflict of Interest

The authors declare that the research was conducted in the absence of any commercial or financial relationships that could be construed as a potential conflict of interest.

## Publisher’s Note

All claims expressed in this article are solely those of the authors and do not necessarily represent those of their affiliated organizations, or those of the publisher, the editors and the reviewers. Any product that may be evaluated in this article, or claim that may be made by its manufacturer, is not guaranteed or endorsed by the publisher.
